# Association of dietary inflammatory index and metabolic syndrome in the elderly over 55 years in Northern China

**DOI:** 10.1017/S0007114521004207

**Published:** 2022-09-28

**Authors:** Ruiqiang Li, Wenqiang Zhan, Xin Huang, Zechen Zhang, Meiqi Zhou, Wei Bao, Qingxia Li, Yuxia Ma

**Affiliations:** 1 Department of Nutrition and Food Hygiene, School of Public Health, Hebei Medical University, Hebei Province Key Laboratory of Environment and Human Health, Shijiazhuang, People’s Republic of China; 2 School of Public Health, Shanghai Jiao Tong University School of Medicine, Shanghai 200025, People’s Republic of China

**Keywords:** Dietary inflammatory index, Elderly, Metabolic syndrome, Nutrition, Diet

## Abstract

We assessed the association between the dietary inflammatory index (DII) and the development of metabolic syndrome in the elderly over 55 years in Northern China. The data of 1936 Chinese adults aged 55 years and over from a community-based neurological disease cohort study from 2018 to 2019 were analysed. Multiple logistic regression and restricted cubic splines regression were used for analysis, and social demographics, lifestyle and health-related factors were adjusted. In the fully adjusted model, the risk of metabolic syndrome increased by 1·28-fold in people with a pro-inflammatory diet. When we divide the metabolic syndrome by its components, high pro-inflammatory diet and hyperglycaemia, TAG, hypertension and abdominal obesity, we failed to observe a significant association between a high pro-inflammatory diet and HDL-cholesterol. However, these associations are moving in the expected direction. At the same time, the results of BMI subgroup analysis showed that with the increase of DII, obese people are at increased risk of metabolic syndrome, hyperglycaemia, high TAG, hypertension and abdominal obesity. Also in overweight people, the increase in DII is accompanied by an increased risk of hyperglycaemia and abdominal obesity. Higher inflammatory diet is related to metabolic syndrome, hypertension, hyperglycaemia, abdominal obesity and hypertriglyceridaemia. Further research is needed to confirm the role of inflammation and diet in the development of metabolic syndrome; however, it is desirable to reduce the dietary components associated with inflammation.

Metabolic syndrome is a common metabolic disorder and has become a public health challenge worldwide^(1)^. It is the simultaneous occurrence of obesity, insulin resistance, atherosclerotic dyslipidaemia (high TAG, LDL-cholesterol) and high blood pressure. It is estimated that 20–30 % of adults suffer from the metabolic syndrome in the world. Metabolic syndrome is associated with an increased risk of type 2 diabetes, non-alcoholic fatty liver, myocardial infarction and stroke. Therefore, it is the main cause of morbidity and mortality worldwide^(2)^.

Metabolic syndrome has become one of the most common chronic diseases in the elderly^(3)^. Some studies have shown that metabolic syndrome and its components are important factors in the progression of atherosclerotic disease in the elderly^(4)^. A metabolic syndrome is a group of metabolic disorders with unknown molecular mechanisms. More and more studies have found that the onset and progression of metabolic syndrome are closely related to inflammation^(5)^. What is more, recent evidence suggests that metabolic syndrome is a consequence of low-grade inflammation^(6,7)^. Diet is one of the main lifestyle-related factors that can regulate the inflammatory process^(8)^. The dietary inflammatory index (DII®) is a tool developed to assess the overall inflammatory potential of an individual’s diet^(9,10)^. It was created by scoring each of the forty-five nutrients and food components that affect CRP, IL-1*β*, IL-4, IL-6, IL-10 and TNF-*α*, using evidence from more than 1900 peer-reviewed journal articles^(10)^.

As the largest developing country in the world, the prevalence of metabolic syndrome is high and rapidly increasing among the elderly population in China^(11)^. Hence, as a result of the huge economic and social burden, metabolic syndrome has become a major challenge for the public healthcare system in China^(12)^. Meanwhile, there are few studies on the relationship between DII and metabolic syndrome in the elderly in China, and there is no consensus on the correlation between them. Thus, this study aimed to evaluate the relationship between DII and the development of the metabolic syndrome and to evaluate its relationship with specific components of metabolic syndrome in the elderly population of northern China.

## Materials and methods

### Study population

**Fig. 1.**
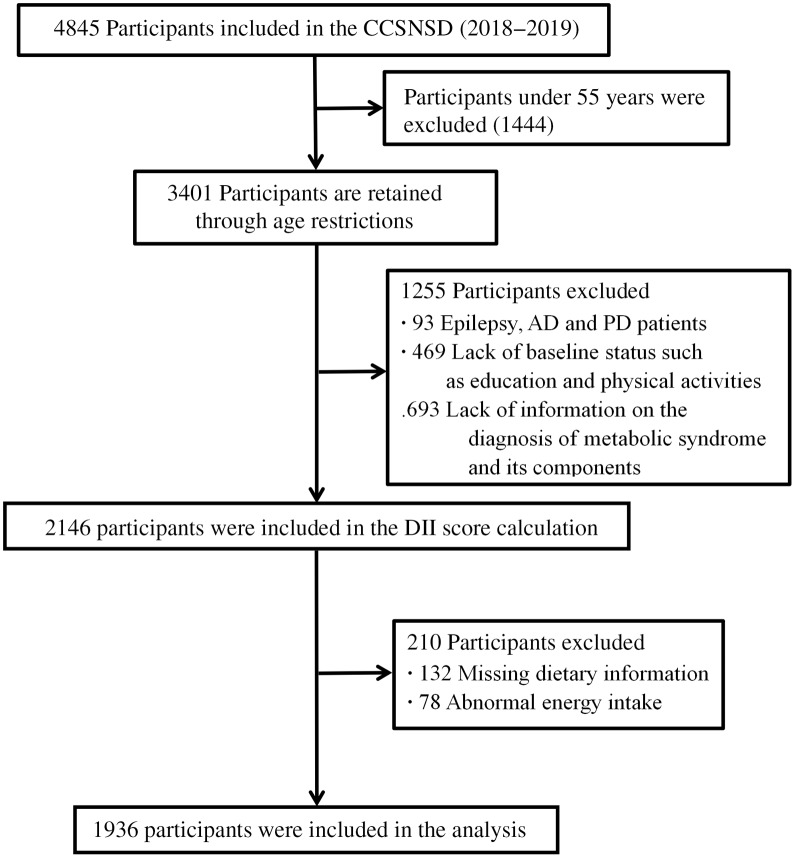
Selection process of subjects.

This study is a cross-sectional study. Participants in the current study were derived from the baseline of the Community Cohort Study of Nervous System Diseases, an ongoing longitudinal study established by the project in 2018–2019, focusing on potential factors related to the risk of three neurological diseases, including epilepsy in patients > 1 year old and Alzheimer’s disease, Parkinson’s disease in people ≥ 55 years old. The project is undertaken by the Institute of Nutrition and Health of the Chinese Center for Disease Control and Prevention, in cooperation with the Center for Disease Control and Prevention. The project uses a multistage random cluster sampling method to draw samples. The protocol of this study was reviewed and approved by the Institutional Review Board of the National Institute for Nutrition and Health (No. 2017020, 6 November 2017). In addition, written informed consent was obtained for each participant before the survey^(13)^. The present study focuses on elderly people over 55 years of age in the cohort study. In the current analysis, we include data from participants who have complete information about diet and are diagnosed with metabolic syndrome. Finally, a total of 1936 participants were involved in the analysis (Fig. 1).

### Metabolic syndrome

We defined the Adult Treatment Group Recommendation III of the Metabolic Syndrome Cholesterol Education Program under national standards, considering the presence of ≥ 3 following components: glucose (≥ 100 mg/dl), TAG (≥ 150 mg/dl), HDL-cholesterol (male < 40 mg/dl and < 50 mg/dl female), BP (systolic blood pressure ≥ 130 mmHg or ≥ 85 mmHg for diastolic blood pressure) and waist circumference (male ≥ 102 cm or ≥ 88 cm for female)^(14)^.

### Assessment of food consumption

Dietary consumption is assessed by a validated semi-quantitative FFQ, covering eighty-one food parameters. Participants were asked about the frequency of habitual consumption the number of each item in the past 12 months and choose from five types of frequencies (daily, weekly, monthly, yearly or never) and consumption in the past 12 months. For consumers, their consumption of each food group or item is calculated based on their reported average consumption frequency and quantity.

### Diet and dietary inflammatory index

There are some studies on the development and verification of DII in detail. Research from more than 1900 peer-reviewed publications forms the basis of DII. The ‘Inflammatory Effect Score’ was created from these peer-reviewed publications for each DII food parameter, based on their impact on inflammatory cytokines. At the same time, standardise DII calculations into a world database with regional representation. This world database includes the dietary consumption of eleven people from all over the world^(10)^. The world database provides standard averages and deviations of all DII food parameters. For each food parameter, create a z-score by subtracting the individual’s estimated intake from the standard average. It is then divided by the world standard deviation and converted to a distribution centred at 0 and bounded between −1 and +1. This value is then multiplied by the inflammatory effect score for each food parameter, and then all food parameters are added together to create an overall DII score. The more positive scores means the more pro-inflammatory diet, the more negative values, the stronger the anti-inflammatory effect^(10)^. The DII food ingredients available in the database include carbohydrates, protein, fat, alcohol intake, fibre, cholesterol, saturated and unsaturated (MUFA) and (PUFA) fatty acids, *n*-3 and *n*-6 PUFA; niacin; vitamins (A, B_1_, B_2_, B_6_, B_12_, C, D and E), Fe, Mg, Zn, Se, folic acid, *β*-carotene and caffeine. DII scores range from negative tail to positive tail, more negative values indicate anti-inflammatory properties and corrected scores indicate pro-inflammatory properties. Energy-adjusted DII (E-DII) food intake per 1000 calories is used to explain the effect of total intake on energy intake^(10,15–17)^.

### Assessment of Covariates

We adjusted the variables previously identified as potential confounders in the literature. Personal background characteristics include self-reported age (yearly), sex (female or male), education level (illiterate, elementary school, junior high school and above), residence (urban or rural) and employment status (yes or no); health-related variables include tobacco smoking (yes or no), alcohol drinking (yes or no) and physical activity (light, moderate and vigorous).

We used a cut-off value of 28 kilograms per square meter (kg/m^2^) of China’s BMI to determine obesity^(18)^. According to the calculation formula of physical activity level, the weekly accumulated metabolic equivalent (MET) value of the elderly is calculated. Then according to the International Physical Activity Scale evaluation criteria, the level of physical activity of the elderly is divided into light (< 600 MET/week), moderate (600–3000 MET/week) and vigorous (> 3000 MET/week)^(19)^.

### Statistical analysis

Quantitative data are expressed as the median (interquartile range), and qualitative data are expressed as the number (percentage), respectively, according to DII quartile. For continuous measures, the Wilcoxon–Mann–Whitney test or Fisher’s exact compared the change in medians across DII quartiles. *χ*^2^ tests were computed to examine the distribution of categorical covariates across quartiles.

Logistic regression and restricted cubic splines regression to simulate the association between the DII quartiles and the metabolic syndrome and calculated OR and 95 % CI to evaluate the relationship between each component of the metabolic syndrome and the DII quartiles. The lowest quartile of the DII score (Q1, reflecting the most anti-inflammatory diet) was used as the reference category. In all cases, we fit a model and adjust the covariates mentioned above. Schoenfeld residuals are used to assess the risk ratio of the model. To optimise the robustness of the statistical test, we conducted a subgroup analysis of BMI (< 18·5, 18·5–24·0,24–28, ≥ 28 kg/m^2^). Statistical analyses were all performed with R 3·6·0 software (http://www.R-project.org, The R Foundation). A two-sided *P*-value < 0·05 was considered to indicate statistical significance.

## Results

Baseline characteristics of the study population are presented by quartiles of the DII score. The DII score ranges from −5·24 to 5·59. The characteristics of the 1936 participants included in this study are shown in Table 1. Among the baseline characteristics, only the differences in age and BMI in the different quartiles of DII were statistically significant. (*P* < 0·05) Participants with highly pro-inflammatory diets (Q4) had higher levels of TAG (mg/dl), HDL-cholesterol (mg/dl) and systolic BP (mmHg).

**Table 1.** Baseline characteristics and components of metabolic syndrome of the community cohort study of nervous system diseases (CCSNSD) project population across quartiles of the DII score(Frequency and percentages; median values and interquartile range)

	Quartile 1 (*n* 483)		Quartile 2 (*n* 484)		Quartile 3 (*n* 485)		Quartile 4 (*n* 484)		
Characteristic	Frequency	%	Frequency	%	Frequency	%	Frequency	%	*P*-value
Age (years)									< 0·001
Median	68		65		63		64		
IQR	64·0–73·0		60·0–70·0		59·0–68·0		60·0–69·0		
Sex									0·938
Male	189	39·1	180	37·2	183	37·7	184	38·0	
Female	294	60·9	304	62·8	302	62·3	300	62·0	
BMI (kg/m^2^)									
BMI < 18·50 (underweight)	9	1·9	22	4·5	13	2·7	12	2·5	0·010
18·50 ≤ BMI < 24·00 (normal weight)	165	34·2	204	42·1	202	41·6	174	36·0	
24·00 ≤ BMI < 28·00 (overweight)	203	42·0	185	38·2	180	37·1	197	40·7	
BMI ≥ 28·00 (obese)	106	21·9	73	15·1	90	18·6	101	20·9	
Residence									0·520
Urban	126	26·1	140	28·9	141	29·1	125	25·8	
Rural	357	73·9	344	71·1	344	70·9	359	74·2	
Employment									0·174
No	427	88·4	409	84·5	406	83·7	415	85·7	
Yes	56	11·6	75	15·5	79	16·3	69	14·3	
Education									0·089
Illiteracy	122	25·3	133	27·5	108	22·3	118	24·4	
Primary school	174	36·0	158	32·6	150	30·9 %	147	30·4	
Junior high school/above	187	38·7	193	39·9	227	46·8	219	45·2	
Tobacco smoking									0·485
No	399	82·6	407	84·1	418	86·2	405	83·7	
Yes	84	17·4	77	15·9	67	13·8	79	16·3	
Physical activities									0·296
Light (<600 MET/week)	139	28·8	137	28·3	135	27·8	135	27·9	
Moderate (600–3000 MET/week)	240	49·7	236	48·8	235	48·5 %	233	48·1	
Vigorous (>3000 MET/week)	104	21·5	111	22·9	115	23·7	116	24·0	
	Median	IQR	Median	IQR	Median	IQR	Median	IQR	
Glucose (mg/dl)	5·60	5·14–6·40	5·57	5·11–6·28	5·49	5·05–6·28	5·60	5·07–6·26	0·268
TAG (mg/dl)	1·48	1·06–2·08	1·35	0·95–1·90	1·35	0·99–1·98	1·36	1·02–1·98	0·012
HDL-cholesterol (mg/dl)	1·24	1·04–1·49	1·33	1·08–1·59	1·30	1·10–1·56	1·26	1·06–1·51	0·012
Systolic BP (mmHg)	135	125·0–147·0	132	121·0–144·0	132·5	122·0–143·0	134	122·0–145·0	0·007
Diastolic BP (mmHg)	82	75·0–89·0	81	75·0–90·0	82	75·0–89·0	81·8	75·0–89·0	0·977
Waist circumference (cm)	90	84·0–97·1	90	82·0–97·0	89	83·2–96·0	90	84·0–96·2	0·489

DII, dietary inflammatory index; MET, metabolic equivalent.

Quartile 1: −5·24–0·81; Quartile 2: 0·81–1·81; Quartile 3: 1·81–2·54; Quartile 4: 2·54–5·59.

The relationship between the metabolic syndrome as a whole and its components and DII quartile is presented in Table 2. In the fully adjusted model, the risk of metabolic syndrome increased by 1·28-fold in people with a pro-inflammatory diet (OR_Q4 *v.* Q1_ = 1·28; 95 % CI: 1·08, 1·56; *P*_linear-trend_ = 0·03). When we divide the metabolic syndrome by its components, high pro-inflammatory diet and hyperglycaemia (OR_Q4 *v.* Q1_ = 1·23; 95 % CI: 1·05, 1·49; *P*_linear-trend_ = 0·04), TAG (OR_Q4 *v.* Q1_ = 1·39; 95 % CI: 1·12, 1·65; *P*_linear-trend_ = 0·02), hypertension (OR_Q4 *v.* Q1_ = 1·28; 95 % CI: 1·09, 1·51; *P*_linear-trend_ = 0·03) and abdominal obesity (OR_Q4 *v.* Q1_ = 1·59; 95 % CI: 1·18, 2·06; *P*_linear-trend_ = 0·01). We failed to observe a significant association between a high pro-inflammatory diet and HDL-cholesterol. However, these associations are moving in the expected direction. There is a potential increase in the risk of HDL-cholesterol observed in the pro-inflammatory diet quartile, but the difference is not significant. Restricted cubic splines regression also shows that DII is positively and non-linearly correlated with metabolic syndrome, hyperglycaemia, high TAG, hypertension and abdominal obesity (*P*_nonlinear-trend_ < 0·05) (online Supplementary Fig. 1).

**Table 2.** OR (95 % CI) for metabolic syndrome and its components in the community cohort study of nervous system diseases (CCSNSD) project population across quartiles of the DII score* (Odds ratio and 95 % confidence intervals)

DII	Metabolic syndrome†		Hyperglycaemia‡		TAG§		HDL-cholesterol||		Hypertension¶		Abdominal obesity**	
	OR	95 % CI	OR	95 % CI	OR	95 % CI	OR	95 % CI	OR	95 % CI	OR	95 % CI
Quartile 1 (*n* 483)	1·00 (Ref)		1·00 (Ref)		1·00 (Ref)		1·00 (Ref)		1·00 (Ref)		1·00 (Ref)	
Quartile 2 (*n* 484)	1·07	0·69, 1·35	1·05	0·76, 1·23	1·13	0·69, 1·33	0·86	0·63, 1·09	1·03	0·68, 1·19	1·21	1·06, 1·68
Quartile 3 (*n* 485)	1·16	0·73, 1·46	1·13	0·98, 1·37	1·21	0·89, 1·36	0·95	0·65, 1·16	1·17	0·65, 1·32	1·48	1·12, 1·89
Quartile 4 (*n* 484)	1·28	1·08, 1·56	1·23	1·05, 1·49	1·39	1·12, 1·65	1·08	0·73, 1·26	1·28	1·09, 1·51	1·59	1·18, 2·06
*P*_trend_	0·03		0·04		0·02		0·39		0·03		0·01	

* Multiple linear regression was used. All models were adjusted for age, sex, educational level, residence, employment status, tobacco smoking and physical activity. DII, dietary inflammatory index; Ref, reference.

† Metabolic syndrome was defined following the National Cholesterol Education Program Adult Treatment Panel III recommendations, which consider the presence of ≥ 3 of the following components shown in footnotes 3–7.

‡ Hyperglycaemia > 100 mg/dl.

§ Hypertriglyceridaemia > 150 mg/dl.

|| HDL-cholesterol: < 40 mg/dl in males or < 50 mg/dl in females.

¶ Hypertension: systolic > 130 mmHg or diastolic > 85 mmHg.

** Abdominal obesity > 102 cm for males or > 88 cm for females.

Table 3 shows the results of the stratified analysis grouped by BMI. In different stratified groups, compared with the least pro-inflammatory diet group (Q1), the DII pro-inflammatory diet group has a different degree of risk in metabolic syndrome and its different components. In the group of overweight, highly pro-inflammatory diets were associated with hyperglycaemia (OR_Q4 *v.* Q1_ = 1·18; 95 % CI: 1·05, 1·62; *P*_linear-trend_ = 0·02), hypertension (OR_Q4 *v.* Q1_ = 1·35; 95 % CI: 1·13, 1·96; *P*_linear-trend_ = 0·01) and abdominal obesity (OR_Q4 *v.* Q1_ = 2·28; 95 % CI: 1·36, 3·12; *P*_linear-trend_ = 0·008). In the obese category, the risk of metabolic syndrome of the most pro-inflammatory diet is 2·18 times that of the least pro-inflammatory diet group (OR_Q4 *v.* Q1_ = 2·15; 95 % CI: 1·08, 3·28; *P*_linear-trend_ = 0·02). Immensely pro-inflammatory diets are correlated with hyperglycaemia (OR_Q4 *v.* Q1_ = 1·42; 95 % CI: 1·15, 1·92; *P*_linear-trend_ = 0·006), TAG (OR_Q4 *v.* Q1_ = 1·56; 95 % CI: 1·06, 2·32; *P*_linear-trend_ = 0·03), hypertension (OR_Q4 *v.* Q1_ = 1·36; 95 % CI: 1·12, 2·32; *P*_linear-trend_ = 0·009) and abdominal obesity (OR_Q4 *v.* Q1_ = 2·01; 95 % CI: 1·20, 3·06; *P*_linear-trend_ = 0·006). At the same time, the results grouped by sex showed that there is no significant gender difference in the correlation between DII and metabolic syndrome (online Supplementary Table 1).

**Table 3.** Subgroup analysis of association between dietary inflammatory index and sex hormones among different BMI groups in CCSNSD 2017–2018 (Odd ratio and 95 % confidence intervals)

Dietary Inflammatory Index Group	Dyslipidaemia											
	Metabolic syndrome (*n* 828)		Hyperglycaemia (*n* 975)		TAG (*n* 671)		HDL-cholesterol (*n* 773)		Hypertension (*n* 1270)		Abdominal obesity (*n* 786)	
	OR	95 % CI	OR	95 % CI	OR	95 % CI	OR	95 % CI	OR	95 % CI	OR	95 % CI
BMI < 18·50 (underweight)												
Quartile 1 (*n* 9)	1·00 (Ref)		1·00 (Ref)		1·00 (Ref)		1·00 (Ref)		1·00 (Ref)		1·00 (Ref)	
Quartile 2 (*n* 22)	1·12	0·39, 1·89	0·59	0·25, 1·13	1·05	0·67, 1·16	0·87	0·35, 1·36	0·92	0·57, 1·32	1·08	0·39, 2·87
Quartile 3 (*n* 13)	1·39	0·58, 2·03	1·09	0·35, 1·96	1·12	0·86, 1·39	1·15	0·86, 1·67	1·25	0·81, 2·32	1·35	0·60, 3·12
Quartile 4 (*n* 12)	1·62	0·67, 2·86	0·63	0·32, 3·02	1·19	0·82, 1·56	1·08	0·62, 1·58	1·58	0·93, 2·96	1·46	0·82, 3·69
*P*-trend	0·52		0·49		0·23		0·61		0·42		0·16	
18·50 ≤ BMI < 24·00 (normal weight)												
Quartile 1 (*n* 165)	1·00 (Ref)		1·00 (Ref)		1·00 (Ref)		1·00 (Ref)		1·00 (Ref)		1·00 (Ref)	
Quartile 2 (*n* 204)	1·05	0·62, 1·63	1·03	0·67, 1·62	0·86	0·49, 1·31	0·69	0·46, 1·21	1·06	0·69, 1·56	1·23	0·72, 2·08
Quartile 3 (*n* 202)	1·09	0·56, 1·56	1·25	0·79, 1·93	1·13	0·49, 1·37	1·05	0·68, 1·63	0·72	0·43, 1·19	1·45	0·86, 2·39
Quartile 4 (*n* 174)	1·16	0·72, 1·85	1·29	0·85, 1·98	1·28	0·62, 1·59	1·26	0·78, 2·02	1·12	0·67, 1·75	1·08	0·60, 1·76
*P*_trend_	0·83		0·37		0·63		0·17		0·32		0·57	
24·00 ≤ BMI < 28·00 (overweight)												
Quartile 1 (*n* 203)	1·00 (Ref)		1·00 (Ref)		1·00 (Ref)		1·00 (Ref)		1·00 (Ref)		1·00 (Ref)	
Quartile 2 (*n* 185)	1·09	0·73, 1·68	1·03	0·53, 1·25	1·15	0·57, 1·52	0·86	0·58, 1·26	0·82	0·53, 1·26	1·78	1·12, 2·90
Quartile 3 (*n* 180)	1·15	0·59, 2·23	1·12	0·64, 1·36	1·26	0·57, 1·90	1·01	0·62, 1·34	1·12	1·03, 1·26	2·02	1·28, 3·19
Quartile 4 (*n* 197)	1·23	0·67, 2·41	1·18	1·05, 1·62	1·67	0·79, 2·16	1·12	0·73, 1·49	1·35	1·13, 1·96	2·28	1·36, 3·62
*P*trend	0·57		0·02		0·19		0·25		0·01		0·008	
BMI ≥ 28·00 (obese)												
Quartile 1 (*n* 106)	1·00 (Ref)		1·00 (Ref)		1·00 (Ref)		1·00 (Ref)		1·00 (Ref)		1·00 (Ref)	
Quartile 2 (*n* 73)	1·08	0·96, 2·05	1·06	0·76, 1·52	1·15	0·65, 1·96	1·03	0·52, 1·86	0·76	0·39, 1·47	1·26	1·05, 1·89
Quartile 3 (*n* 90)	1·63	1·06, 2·18	1·23	1·08, 1·68	1·29	0·75, 2·02	1·19	0·48, 1·65	1·08	0·62, 2·06	1·52	1·16, 2·35
Quartile 4 (*n* 101)	2·15	1·08, 3·28	1·42	1·15, 1·92	1·56	1·06, 2·32	1·35	0·97, 2·56	1·36	1·12, 2·32	2·01	1·20, 3·06
*P*_trend_	0·02		0·006		0·03		0·26		0·009		0·006	

## Discussion

This study aims to assess the association between DII scores and the incidence of metabolic syndrome in samples of healthy elderly people from northern China. Compared with participants on a highly anti-inflammatory diet, people on a highly inflammatory diet had a 1·28 times the risk of developing metabolic syndrome. In addition, we have observed that consumers on a highly inflammatory diet are at higher risk of hypertriglyceridaemia, hypertension, hyperglycaemia and abdominal obesity. At the same time, the results of BMI subgroup analysis showed that with the increase of DII, obese people are at increased risk of metabolic syndrome, hyperglycaemia, high TAG, hypertension and abdominal obesity. Also in overweight people, the increase in DII is accompanied by an increased risk of hyperglycaemia and abdominal obesity.

The possible reason for the results of the study is that we found that a highly pro-inflammatory diet is characterised by an unhealthy diet. Participants on a high-inflammatory diet consumed more SFA, which increased TAG reserves in adipose tissue by activating the inflammatory response^(20)^. They also consume fewer antioxidants, which can change the redox balance, cause endothelial damage and further increase the inflammatory response. Red meat is also often eaten; this is related to an increase in soluble adhesion molecules^(21,22)^. The consumption of soft drinks (fructose) is related to the activation of different mechanisms that promote inflammation. Fructose promotes the activation of oxidative stress and NF-κB, thereby inducing stress response through liver and lipid metabolism disorders^(23)^.

The relationship between metabolic syndrome and dietary inflammation index may be related to dietary patterns. Eating patterns are one of the best ways to assess the relationship between diet and disease. In different studies, the positive effects of healthy eating patterns in preventing cancer, diabetes and obesity have been proven. Recently, it has been shown that the anti-inflammatory DII score is positively correlated with some healthy eating patterns, such as DASH (Eat Method to Prevent High Blood Pressure), Alternative Healthy Eating Index and Healthy Eating Index-2010^(24–26)^.

We found an association between DII and the three components of metabolic syndrome: hypertriglyceridaemia, hypertension, abdominal obesity and blood sugar levels; we did not observe an association with HDL-cholesterol. The specific components of the metabolic syndrome and their relationship with DII were analysed only in cross-sectional studies. In the USA, a cross-sectional study of the police population observed a higher prevalence of hyperglycaemia in participants with pro-inflammatory DII^(27)^. This is consistent with our research results. At the same time, a study was conducted in eight cities in China to observe the association between DII and the risk of hypertension, and the strength of the association was similar to our research results^(28)^. In addition, a study conducted among Polish adults found an association between DII and risk of abdominal obesity in men (OR_Q4 *v.* Q1_ = 1·65; 95 % CI: 1·01, 2·69)^(28)^. We observed a similar correlation. Participants with the highest DII levels had a 1·59-fold increased risk of abdominal obesity (OR_Q4 *v.* Q1_ = 1·59; 95 % CI: 1·18, 2·06; *P*_linear-trend_ = 0·01). Our results did not find an association between DII and HDL-cholesterol. This requires further research to assess the association of DII with specific components of the metabolic syndrome, to assess the robustness of our research and to better understand the underlying mechanisms of inflammatory diets affecting health.

As far as we know, the present study is the first to explore the association between DII and metabolic syndrome in the elderly over 55 years in China. All of the participants recruited into the cohort are from the same province, their income, education and serological indicators are not representative. Thus, the dietary intake observed in the cohort is not necessarily similar to the national elderly. Nonetheless, our research still captured enough variation in the diet to observe a link between quartile DII and metabolic syndrome. There are several limitations in our study. First, the dietary consumption level is estimated based on the FFQ covering the past 12 months, which may have a certain recall bias. Another potential limitation is that among the forty-five food parameters, only twenty-two food parameters can be used in the DII calculation in this article, and the estimation of the potential of dietary inflammation may be biased. Although we carefully adjusted some covariates in the data analysis, there may still be residual confusion^(10,29)^. In addition, the cross-sectional nature of our research does not allow us to draw any causal conclusions.

### Conclusion

Our results showed that higher inflammatory diet is related to metabolic syndrome, hypertension, hyperglycaemia, abdominal obesity and hypertriglyceridaemia. The trend was also evident in overweight and obese people. Further research is needed to confirm the role of inflammation and diet in the development of metabolic syndrome; however, it is desirable to reduce the dietary components associated with inflammation.

## Supplementary material

For supplementary material accompanying this paper visit https://doi.org/10.1017/S0007114521004207. click here to view supplementary material
