# Supplementary material for: Association of dietary inflammatory index and metabolic syndrome in the elderly over 55 years in Northern China
Source: Br J Nutr. 2021 Oct 18;128(6):1082–9. doi: 10.1017/S0007114521004207 (PMC9381302; doi:10.1017/S0007114521004207)
Supplement: Supplementary file 1 [file S0007114521004207sup001.docx]

**Supplementary materials**

**
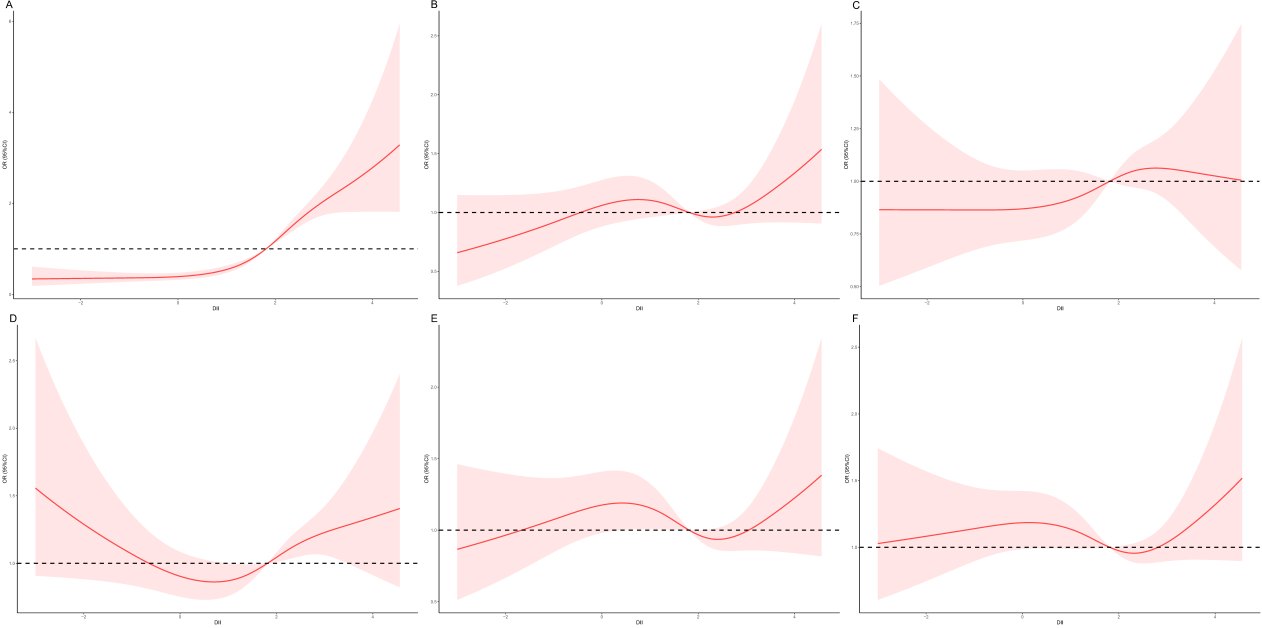
**

Supplementary Figure 1 Restricted cubic splines for the nonlinear relationship between the risk of Metabolic syndrome and its components and increased DII scores. (A),Metabolic syndrome;(B),Hyperglycemia;(C),Triglycerides;(D),HDL-C;(E)Hypertension;(F),Abdominal obesity. RCS models were adjusted for age, sex, educational level, residence, employment status, tobacco smoking, and physical activity. The solid line displays the odds ratio (OR), and the dashed line represents the 95% confdence interval (CI).

Supplementary Table 1 Subgroup analysis of association between Dietary Inflammatory Index and sex hormones among different sex groups in CCSNSD 2017-2018

| Dietary Inflammatory Index Group |  | | Dyslipidemia | |  | |
| --- | --- | --- | --- | --- | --- | --- |
|  | Metabolic syndrome (n=828) | Hyperglycemia (n=975) | Triglycerides (n=671) | HDL-C (n=773) | Hypertension (n=1270) | Abdominal obesity (n=786) |
| Male |  |  |  |  |  |  |
| Quartile 1（n=189） | 1.00 (Ref) | 1.00 (Ref) | 1.00 (Ref) | 1.00 (Ref) | 1.00 (Ref) | 1.00 (Ref) |
| Quartile 2（n=180） | 1.09(0.86-1.76) | 1.05(0.82-1.37) | 1.02(0.78-1.29) | 0.78(0.37-1.08) | 0.91(0.49-1.32) | 1.19(1.02-1.52) |
| Quartile 3（n=183） | 1.21(0.98-1.89) | 1.28(1.02-1.91) | 1.16(0.92-1.45) | 1.09(0.78-1.32) | 1.17(0.82-1.69) | 1.37(1.12-1.59) |
| Quartile 4（n=184） | 1.65(1.08-2.06) | 1.76(1.13-2.15) | 1.37(1.02-1.68) | 1.15(0.92-1.61) | 1.36(1.08-1.82) | 1.52(1.18-1.96) |
| P-trend | 0.04 | 0.03 | 0.02 | 0.19 | 0.009 | 0.003 |
| Female |  |  |  |  |  |  |
| Quartile 1（n=294） | 1.00 (Ref) | 1.00 (Ref) | 1.00 (Ref) | 1.00 (Ref) | 1.00 (Ref) | 1.00 (Ref) |
| Quartile 2（n=304） | 0.92(0.59-1.37) | 1.09(0.83-1.29) | 0.98(0.67-1.22) | 0.80(0.45-1.12) | 1.02(0.72-1.30) | 1.10(0.92-1.35) |
| Quartile 3（n=302） | 1.17(0.79-1.48) | 1.25(1.03-1.58) | 1.15(0.89-1.39) | 1.06(0.69-1.31) | 1.19(1.01-1.48) | 1.31(1.09-1.52) |
| Quartile 4（n=300） | 1.36(1.08-1.57) | 1.68(1.09-2.06) | 1.39(1.05-1.72) | 1.19(0.98-1.75) | 1.35(1.13-1.62) | 1.63(1.18-2.02) |
| P-trend | 0.02 | 0.01 | 0.04 | 0.12 | 0.008 | 0.002 |
